# Surgical treatment for basilar invagination with irreducible atlantoaxial dislocation: transoral atlantoaxial reduction plate fixation vs occipitocervical fixation

**DOI:** 10.1186/s12891-020-03838-6

**Published:** 2020-12-08

**Authors:** Xiaobao Zou, Bieping Ouyang, Haozhi Yang, Binbin Wang, Su Ge, Yuyue Chen, Ling Ni, Shuang Zhang, Hong Xia, Jingcheng Yang, Xiangyang Ma

**Affiliations:** 1 The First School of Clinical Medicine, Southern Medical University, No.1838 North of Guangzhou Road, Guangzhou, 510515 People’s Republic of China; 2 Department of Orthopedics, General Hospital of Southern Theatre Command of PLA, No.111 Liuhua Road, Guangzhou, 510010 People’s Republic of China; 3 Department of Spinal Surgery, Nanfang Hospital, Southern Medical University, No.1838 North of Guangzhou Road, Guangzhou, 510515 People’s Republic of China

**Keywords:** Basilar invagination, Irreducible atlantoaxial dislocation, Transoral approach, Transoral atlantoaxial reduction plate, Occipitocervical fusion, Internal fixation

## Abstract

**Background:**

Transoral atlantoaxial reduction plate (TARP) fixation or occipitocervical fixation (OF) is an effective treatment for basilar invagination (BI) with irreducible atlantoaxial dislocation (IAAD). But, all current clinical studies involved a single surgical procedure. The clinical effects of TARP and OF operation for BI with IAAD have yet to be compared. We therefore present this report to compare the treatment of TARP and OF procedure for BI with IAAD.

**Methods:**

Fifty-six patients with BI with IAAD who underwent TARP or OF operation from June 2011 to June 2017 were retrospectively analyzed. Among these, 35 patients underwent TARP operation (TARP group), and 21 patients underwent OF operation (OF group). We compared the difference of clinical, radiological, and surgical outcomes between the TARP and OF groups postoperatively.

**Results:**

Compared with OF group, the operative time and blood loss in TARP group were lower. There was no statistical difference in the atlantodental interval (ADI), clivus canal angle (CCA), cervicomedullary angle (CMA), distance between the top of the odontoid process and the Chamberlain line (CL) and Japanese Orthopaedic Association (JOA) score between the TARP and OF groups preoperatively, but the improvements of these parameters in the TARP group were superior to those in the OF group postoperatively. The fusion rates were higher in the TARP group than those in the OF group at the early stage postoperatively.

**Conclusions:**

TARP and OF operations are effective surgical treatment for BI with IAAD, but the performance of reduction and decompression and earlier bone fusion rates of TARP procedure are superior to those of OF.

## Background

Basilar invagination (BI) is a relatively common congenital or developmental anomaly of the craniocervical junction characterised by prolapse of the odontoid process into the foramen magnum, generally accompanied by irreducible atlantoaxial dislocation (IAAD). The condition may result in spinal cord and brainstem (medullary) compression, thus leading to severe neurological injury. Persistent serious compression can cause respiratory disturbance, with high mortality rates [1, 2]. As a result of abnormal structure, complicated anatomy, deep position, and proximity to the vertebral artery and medulla, surgical treatment of BI with IAAD is difficult and poses a high risk [3, 4]. Transoral atlantoaxial reduction plate (TARP) fixation [5–8] and occipitocervical fixation (OF) [9–13] are two frequently-used surgical approaches for treating BI with IAAD. Several current clinical studies [5–13] have confirmed the effectiveness of TARP and OF operations, but all involved a single surgical procedure. Therefore, we conducted a study to compare the treatment of two surgical procedures for BI with IAAD.

## Methods

### Patients and data collection

In this retrospective study, we enrolled patients diagnosed with BI with IAAD undergoing a TARP or OF operation between June 2011 and June 2017. Skull traction was performed in all patients for 1 week preoperatively, and no sings of reduction was found in all cases. All patients underwent transoral anterior release before TARP or OF procedure. Then, patients who had oropharyngeal diseases or small oral space underwent OF procedure, otherwise, both TARP and OF procedures could be used. Exclusion criteria were (1) previous surgery such as posterior decompression of the foramen magnum or decompression of the anterior arch of C1; (2) a cervical intraspinal or osseous tumour; (3) severe osseous anomalies of the craniovertebral junction, hindering measurement; and (4) less than 12 months of follow-up.

According to our criteria, a total of 56 patients were included in the study. Thirty-five patients underwent TARP surgery (TARP group), and 21 cases were treated with OF surgery (OF group). Each patient’s clinical data were reviewed carefully. Data collection included demographics, clinical manifestations, radiological parameters, surgical details such as internal fixation technique, intraoperative blood loss, operative time, and complications.

### Surgical procedures

Preoperative preparation: All patients were required to gargle 3–6 times per day with vinegar chlorhexidine 0.02% for 3 days before surgery. A preoperative professional dental cleaning was performed. All patients received intravenous broad-spectrum antibiotics 30 min before operation.

#### TARP

This surgical technique has been previously described by Yin et al. [5, 6]. The main points of the technique were introduced below.

Under general anaesthesia with transnasopharyngeal intubation, the patient was positioned supine with skull traction of 4–12 kg. After conventional oral cleaning, the face and oropharyngeal cavities were disinfected repeatedly with an iodophor, normal saline, hydrogen peroxide, and chlorhexidine before placement of the surgical drapes. Posterior pharyngeal wall was incised lengthwise by 3–4 cm to expose the anterior structure of the C1-C2 after subperiosteal exfoliation of the mucosa and muscle. The anterior hyperplastic scar tissue, articular capsule and intraarticular cartilage were then thoroughly resected to loosen atlantoaxial joint. If the release was unsatisfactory, the extent of the release was expanded, even if it was necessary to cut the odontoid process and alar ligaments. Then, the articular surface of the bilateral mass joints was ground off with a high-speed bur in preparation for bone grafting. According to the measurement of the distance between the entry points of the bilateral C1 lateral mass screws, an appropriate size TARP was selected. Then, two screws were used to fix the TARP to the anterior surface of the atlas. After a temporary reduction screw was implanted into the C2 vertebral body through the open slip section in the central part of the TARP, the upper arm of a reduction forceps was installed to hold the crossbar of the TARP and the inferior arm hold the temporary reduction screw. Then, the forceps handles were closed to distract the C1-C2 joint for lengthways reduction, and the nut on the upper arm was then turned to push C1 back towards the odontoid process for horizontal reduction. After the reduction was confirmed to be satisfactory on intraoperative radiographs, retropedicle screws or vertebral screws were implanted in C2 to solidly fix the TARP, and the temporary reduction screw was then removed. An intraoperative radiograph further confirmed the location of the screws and the reduction of the C1-C2 joint. After the autogenous iliac bone was grafted into the bilateral lateral mass joints space, the muscular and mucosal layers of the wound were closed.

#### OF

Under general anaesthesia with transnasopharyngeal intubation, the patient was positioned supine first with skull traction of 4–12 kg. Transoral anterior release procedure, as described in TARP operation, was performed primarily. Subsequently, the patient was changed to prone position with the same weight skull traction. The skin was incised longitudinally for about 8 cm along the posterior midline of the neck from the occipital to the C2 spinous process. The occipital bone, the posterior arch of C1, and the lateral mass of C2 were exposed by subperiosteal dissection in both directions, and the attachment of the C2 spinous process was retained. The C2 bilateral pedicle screw paths were prepared, and then screws were placed after tapping. If the pedicle screws were unsuitable for C2 fixation due to anatomical variations, the translaminar screws or pars screws were used instead for C2 fixation. Then an occipital plate was placed. The rods were then contoured and loaded onto the C2 screw heads and occipital plate on both sides and locked. The C1 posterior arch, the C2 lamina, and the lower part of the occipital plate were ground off with a high-speed bur to prepare the bone-graft bed. The autogenous iliac cancellous bone was grafted. After a negative pressure drainage tube was placed, the incision was sutured layer by layer.

### Postoperative management

The nasal trachea cannula was removed 24–48 h after operation, and the nasogastric feeding tube was removed in 7–10 days after the wound healing was confirmed by an electronic laryngoscope. Ultrasonic nebulisation and 0.02% chlorhexidine gargling were performed 3–6 times per day for 1 week. All patients received intravenous broad-spectrum antibiotics for 3 days postoperatively.

### Radiological and neurological evaluation

The cervical X-ray radiographs, computed tomography (CT) scans and magnetic resonance imaging (MRI) were obtained before surgery, at discharge and then at each follow-up. Patients were followed up at 3, 6 and 12 months and then once per year or whenever needed. Postoperative surgery-related or nonsurgical-related complications were recorded. Bone fusion was determined by bone bridge formation showed on CT scan. Patients’ neurologic status was assessed using the Japanese Orthopaedic Association (JOA) score before surgery, at discharge and then at each follow-up.

The atlantodental interval (ADI), the clivus canal angle (CCA) and the distance between the top of the odontoid process and the Chamberlain line (CL) were measured on the preoperative, discharge and 3-month follow-up CT images to evaluate the reduction of C1-C2. The ventral compression of the spinal cord and medulla was assessed by the cervicomedullary angle (CMA) measured on the sagittal MRI scans.

### Statistical analysis

Statistical analysis was performed using the SPSS 19.0 software (IBM, Armonk, NY, USA). The comparison between the two groups were performed using the independent-samples *t* test for quantitative data, and the Pearson’s chi-squared test for categorical data. The paired-samples t test was used to analyze any statistical difference between the pre- and postoperative measurements in each group. The level of significance was set at *p* < .05.

## Results

### Clinical and surgical characteristics

The clinical and surgical information are summarized in Table 1. No significant statistical differences were found both in gender ratio and age between the TARP and OF groups. The most frequent clinical symptom was extremities weakness, followed by numbness, occiput and neck pain, dystaxia, dyspnea and the others in both groups. There was no significant difference in the frequencies of symptoms and symptom treatment interval between groups. The rates of concomitant malformation were not different between the two groups.

**Table 1:** The clinical and surgical data of 56 patients

Characteristic	TARP group	OF group	*P*
Number of patients/n	35	21	
Sex/n(%)			.18
Male	14 (40.0%)	9 (42.9%)	
Female	21 (60.0%)	12 (57.1%)	
Age/y (range)	42.5 ± 15.6 (12–70)	38.6 ± 14.2 (14–71)	.35
Symptom presentation/n (%)			
Extremities weakness	34 (97.1%)	19 (90.5%)	.54
Numbness	27 (77.1%)	13 (61.9%)	.33
Occipital and neck pain	22 (62.9%)	12 (57.1%)	.67
Dystaxia	13 (37.1%)	5 (23.8%)	.30
Dyspnea	5 (14.3%)	2 (9.5%)	.70
Others	11 (31.4%)	6 (28.6%)	.82
Concomitant malformation/n (%)			
Atlas assimilation	29 (82.9%)	18 (85.7%)	.78
Klippel-Feil syndrome	10 (28.5%)	4 (19.0%)	.12
Chiari malformation	5 (14.3%)	2 (9.5%)	.70
Syringomyelia	13 (37.1%)	6 (28.5%)	.51
Symptom treatment interval/months (range)	63.2 ± 50.4 (12–240)	49.9 ± 55.8 (6–240)	.36
Operative time/min (range)	227.6 ± 61.5 (90–420)	325.2 ± 123.4 (150–600)	<.01
Blood loss/ml (range)	123.1 ± 54.9 (50–250)	271.4 ± 142.8 (50–500)	<.01
Postoperative complications/n (%)	3 (8.5%)	1 (4.7%)	.59
Pulmonary infections	1 (2.9%)	0	
Deep vein thromboses	1 (2.9%)	0	
Urinary tract infections	1 (2.9%)	1 (4.7%)	
Follow-up/months	36.6 ± 16.0 (12–72)	41.6 ± 18.1 (12–84)	.29

*TARP* Transoral atlantoaxial reduction plate, *OF* Occipitocervical fixation, *Others* Including dizziness, sleep apnea, tinnitus, hoarseness, dysfunction of excretion

**Fig. 1:**
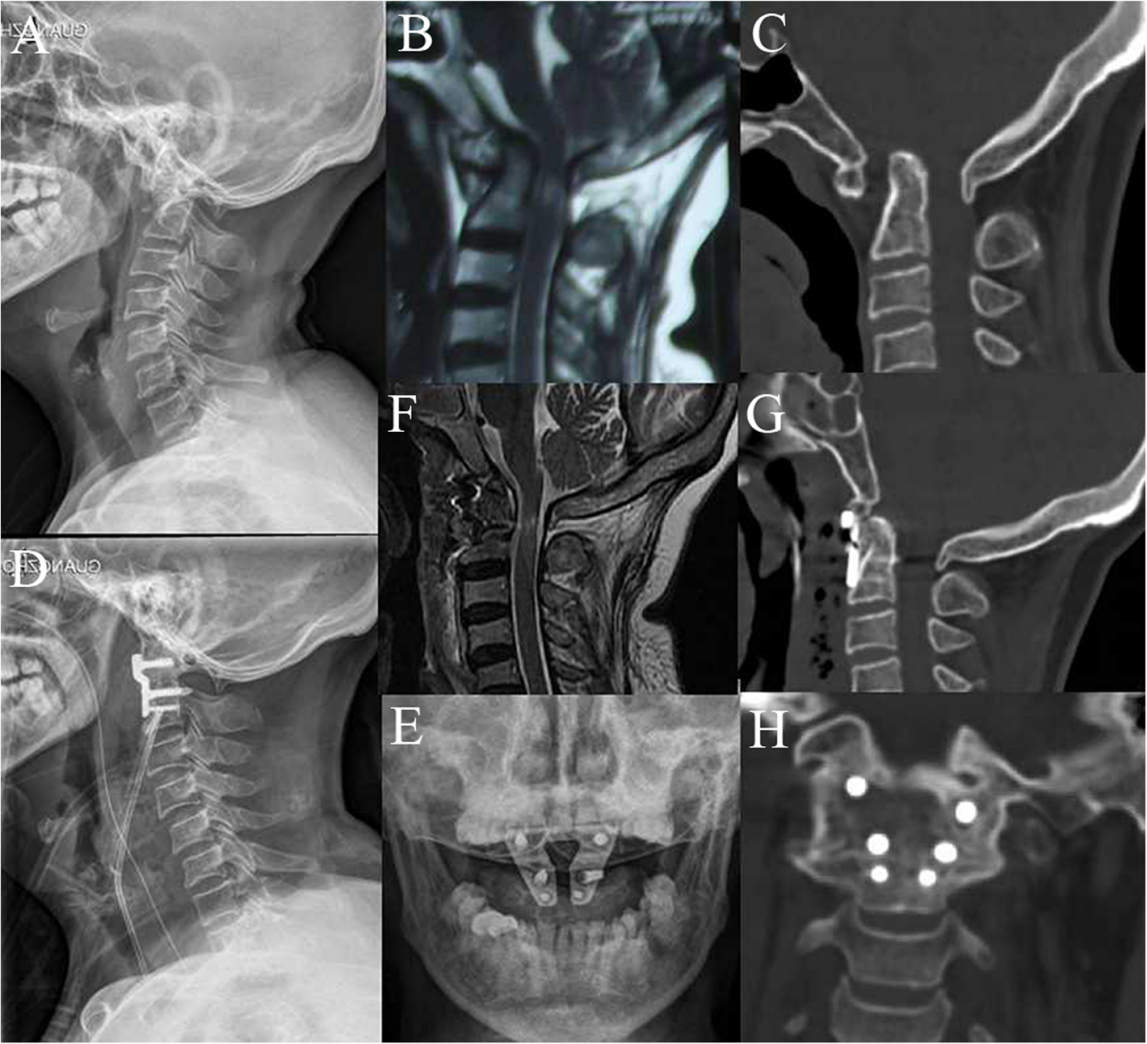
A 64-year-old woman, diagnosed with basilar invagination with atlantoaxial dislocation, underwent a TARP procedure. Images of cervical lateral radiographs (**a**), sagittal MRI (**b**), and sagittal CT scan (**c**) before surgery showed evidence of basilar invagination with atlantoaxial dislocation and compression on the ventral medulla. Cervical radiographs (**d**, **e**) after a TARP operation showed well placement of device. Postoperative sagittal MRI (**f**) and sagittal CT scan (**g**) showed reduction of atlantoaxial dislocation with descent of the odontoid process and decompression on the ventral medulla. CT image (**h**) at 3 months after surgery revealed solid bone fusion

**Fig. 2:**
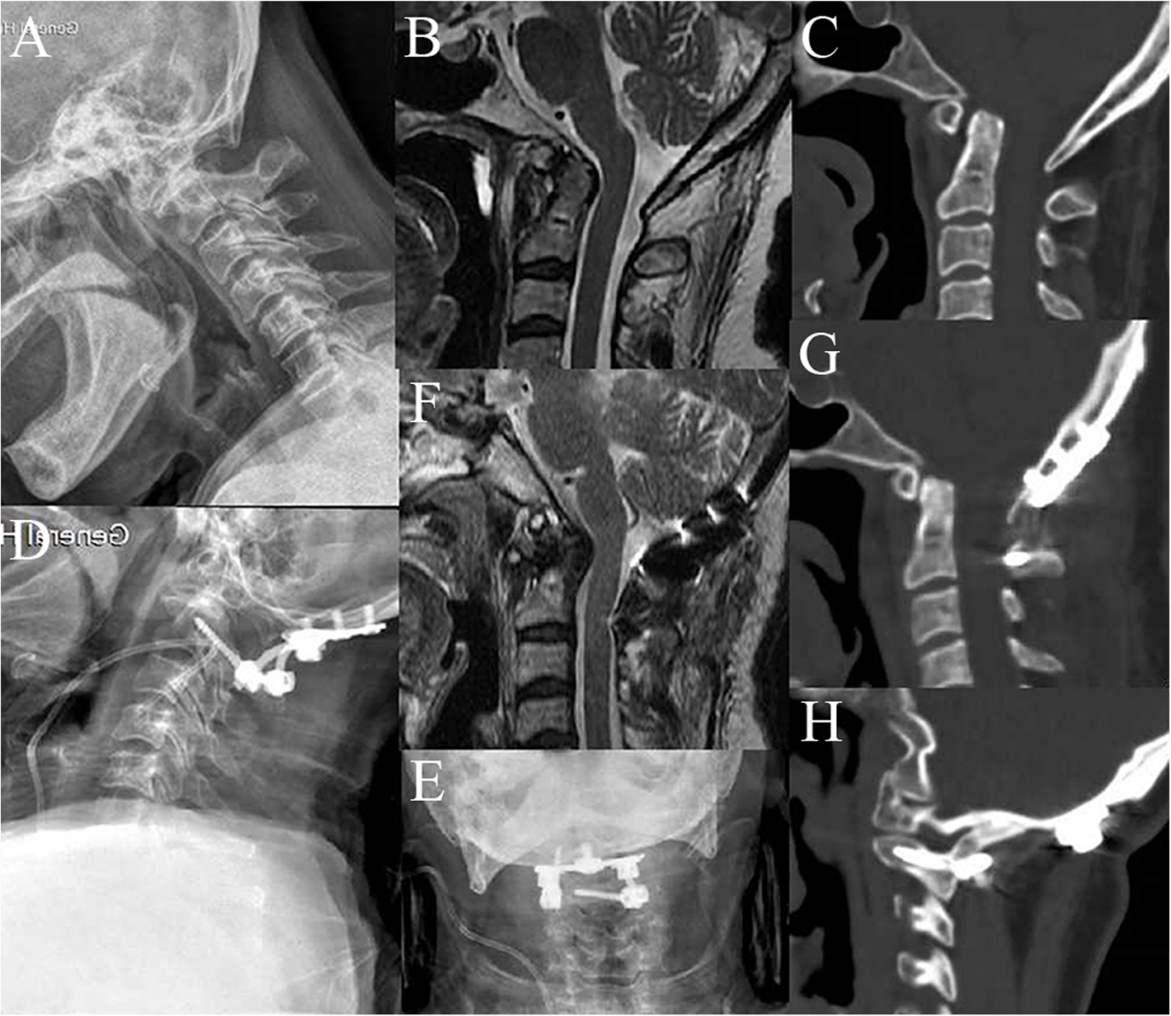
A 62-year-old man, diagnosed with basilar invagination with atlantoaxial dislocation, underwent an OF surgery. Preoperative images of cervical lateral radiographs (**a**), sagittal CT scan (**b**), and sagittal MRI (**c**) showed evidence of basilar invagination with atlantoaxial dislocation and compression on the ventral. Cervical lateral radiographs (**d**, **e**) after an OF operation showed satisfactory location of internal fixation. Postoperative sagittal MRI (**f**) and sagittal CT scan (**g**) showed repositioning of atlantoaxial dislocation and alleviation of compression on the medulla. CT image (**h**) from the 6-month follow-up showed solid bone fusion

All 56 patients underwent successful surgeries (Figs. 1 and 2). No major neurovascular injury occurred during the surgical procedure. A significant statistical difference was found between the TARP (227.6 ± 61.5 min) and OF (325.2 ± 123.4 min) groups for the mean operative time (*p* < .01). The blood loss was significantly lower in the TARP group (123.1 ± 54.9 mL) than that in the OF group (271.4 ± 142.8 mL) (*p* < .01). After surgery, nonsurgical-related complications occurred in three patients (8.5%) in the TARP group and one patient (4.7%) in the OF group (*p* = .59). The average follow-up time of the TARP group was 36.6 ± 16.0 months (range 12–72 months), which had no statistical difference compared with that in the OF group (41.6 ± 18.1 months, range 12–84 months; *p* = .29).

### Radiological and neurological outcomes

These data are summarized in Table 2. No statistical difference was found in the radiological measurements of ADI, CCA, CL, and CMA between the two groups before operation. These parameters in both groups had significant improvements after operation. The ADI and CL in the TARP group were significantly less than those in the OF group at the discharge and at the 3-month follow-up (*p* < .01). The CCA and CMA in the TARP group were significantly greater than those in the OF group at the discharge and at the 3-month follow-up (*p* < .01). There was no difference in the JOA score between two groups before operation (*p* = .34). Significant improvement of JOA score was reflected in both groups postoperatively, but the JOA score in two groups had no statistical difference at discharge. However, when analyzing the data at 3-, 6- and 12-month follow-ups, the JOA scores in the TARP group were significantly higher than those in the OF group (*p* < .01; *p* < .01).

**Table 2:** Comparisons of pre- and postoperative radiological and neurological evaluation between the two groups

Items	TARP group	OF group	*P*
ADI/mm			
Before operation	6.3 ± 2.3	5.7 ± 1.9	.31
At discharge	1.9 ± 1.1	2.9 ± 1.2	<.01
3-month follow-up	1.9 ± 1.1	2.9 ± 1.2	<.01
CL/mm			
Before operation	11.1 ± 3.5	10.2 ± 4.8	.43
At discharge	1.0 ± 1.8	3.0 ± 3.1	< .01
3-month follow-up	1.0 ± 1.8	3.0 ± 3.1	< .01
CCA/°			
Before operation	122.1 ± 14.7	123.1 ± 18.0	.82
At discharge	151.5 ± 10.2	139.5 ± 17.2	< .01
3-month follow-up	151.8 ± 10.1	139.6 ± 17.2	< .01
CMA/°			
Before operation	125.4 ± 10.9	130.1 ± 16.4	.20
At discharge	156.4 ± 7.1	145.0 ± 14.2	<.01
3-month follow-up	157.0 ± 6.9	145.3 ± 14.3	<.01
JOA score			
Before operation	10.7 ± 1.5	11.1 ± 1.3	.34
At discharge	12.6 ± 1.3	12.0 ± 1.0	.06
3-month follow-up	13.8 ± 1.0	13.0 ± 0.9	<.01
6-month follow-up	14.3 ± 0.8	13.7 ± 0.6	<.01
12-month follow-up	15.4 ± 1.00	14.5 ± 0.9	<.01
Bone fusion/n (%)			
3-month follow-up	22 (62.9%)	7 (33.3%)	.03
6-month follow-up	30 (85.7%)	13 (61.9%)	.04
12-month follow-up	35 (100%)	20 (95.2%)	.36
Final follow-up	35 (100%)	21 (100%)	NS

*ADI* Atlantodental interval, *CCA* Clivus canal angle, *CL* Distance between the top of the odontoid process and the Chamberlain line, *CMA* Cervicomedullary angle, *JOA* Japanese Orthopaedic Association, *NS* Not significant

The bone fusion rates were higher in the TARP group than those in the OF group at 3- and 6-month follow-ups (*p* < .05; *p* < .05). There was no significant difference in the bone fusion rates between groups at the 12-month follow-up, with 100% of fusion was achieved in the TARP group and 95.2% in the OF group. Finally, one patients (4.7%) in the OF group did not obtain bone fusion at the subsequent follow-up and the final fusions were achieved after a revision surgery with cancellous bone grafting. During the full follow-up period, no signs of re-dislocation or instrument failure and other late complications were documented in both groups.

## Discussion

BI, accompanied by IAAD, invariably results in evolutionary compression of the medulla, leading to severe neurological damage and even death. Surgical treatment is often required for most patients to restore the dislocation, reconstruct stability, relieve clinical symptoms [14]. At present, OF operation is the most popular posterior surgical procedure for treating BI with IAAD using plate-screw-rod instruments [9–13]. The TARP system was designed by Yin et al. in 2004, and it is an effective surgical approach for treating irreducible atlantoaxial dislocation caused by congenital developmental anomaly, tumour, trauma, and so on, completing release, reduction, decompression, fixation, and fusion in one step with an anterior-only approach [15–17]. For treatment of BI with IAAD, the TARP system possesses distinctive advantages, because it can move down the odontoid process from the foramen magnum and achieve reduction to directly relieve the compression anterior to the spinal cord [5, 6].

Several clinical studies have confirmed the effectiveness of TARP and OF operation for the treatment of BI with IAAD, but all involved a single surgical procedure [5–13]. We retrospectively analyzed the clinical profiles of 56 patients diagnosed with BI with IAAD who underwent a TARP or OF operation, with the aim of comparing the two treatments for BI with IAAD.

In this study, patients undergoing TARP or OF operation obtained significant radiological improvement in postoperative ADI, CCA, CL and CMA demonstrating the effect of two surgical approaches for reduction and decompression. Moreover, postoperative JOA scores in all patients obviously improved. The aforementioned result explains that the use of a TARP or OF operation is an effective surgical approach for the treatment of BI with IAAD, consistent with previous studies.

Found in comparative analysis of surgical characteristics, the operative time and blood loss in the TARP group were less than those in the OF group, which might because the TARP operation can be performed with an transoral anterior-only approach [5–8, 15–17], while OF operation often requires concomitant transoral anterior release before the posterior approach is made in order to achieve satisfactory reduction and decompression [9–13]. Otherwise, operative time and blood loss would be increased, leading to a wider range of surgical trauma and a higher risk of infection. Furthermore, the spinal cord might have been injured while in the transforming operative position after transoral anterior release when the atlantoaxial joint was extremely unstable [14].

Although no significant differences were found in the preoperative radiographic measurements of ADI, CCA CL, and CMA between the TARP and OF groups, compared with OF, the improvements of postoperative ADI, CMA, CCA, and CL in the TARP group were superior. This indicates that TARP operation was more effective than OF operation in reducing BI with IAAD, bound up with a more direct method of reduction by TARP than by OF. By using reduction instruments directly in TARP procedures, we were able to separate the atlantoaxial junction and pull the axis with the odontoid process inferiorly and subsequently push C1 backward relative to C2. We thus achieved atlantoaxial reduction, while OF procedures reduced the atlantoaxial junction to allow the axis, with the odontoid process, to descend indirectly via the strength generated by the posterior plate-screw-rod instruments.

Satisfactory neurological improvements were achieved in both the TARP and OF groups after operations. Despite no difference in preoperative JOA scores between two groups at discharge, the JOA score of the TARP group was significantly higher than that in the OF group at the 3-, 6- and 12-month follow-ups. Patients with BI and IAAD suffered from ventral spinal cord compression induced by ascent of the odontoid process. Jiang et al. [18] reported that the craniocervical volume change rate is a positive predictor for evaluating the improvement of postoperative neurological function in patients with BI. The study by Wei et al. [16] confirmed the negative correlation between the CL and the craniocervical volume improvement rate. The postoperative CL in TARP group was significantly less than that in OF group, so that, the function of TARP operation for improving craniocervical volume could be more obvious than that of OF, which could be more beneficial for recovering of impaired spinal nerves.

We also found that the bone fusion rate was much higher at the early stage postoperatively in the TARP group than that in the OF group. It might be related to methods of bone grafting in two procedures. Although, both TARP and OF fixation had a excellent biomechanical stability proved by previous researches [19–21], more pressure was put on bone grafts in TARP procedure than in OF operation, because most bone was grafted into the lateral mass joint space in TARP operation [22], while all bone was grafted on the surfaces of C1 posterior arch, C2 lamina, and lower part of the occipital in OF operation [23].

Wound infection is another concern. Most surgeons select OF fixation mainly because of this complication. TARP fixation might be more possible to cause the occurrence and spread of infection [24–26]. But, with proper preoperative preparation and postoperative care, the complication rate can be reduced. In this study, no patient had wound infection in TARP group.

There are several limitations in this study. Selection bias exists in this study due to the single-center analysis, that hints the requirement of a multicenter, large sample study in the future. Additionally, the present study is retrospective in nature; future prospective studies may better control for follow-up timing intervals and may have the potential to include more standardized outcome measures.

## Conclusions

The TARP and OF operations are effective surgical approaches for treating BI with IAAD. However, the TARP procedure offers a more direct and ideal performance than does OF in reduction and decompression. Moreover, TARP operation could obtain earlier solid bone fusion. The TARP technique may be a superior choice for treatment of BI with IAAD.

## Data Availability

The data used and analyzed during the current study are available in anonymized form from the corresponding author on reasonable request.

## Abbreviations

TARP: Transoral atlantoaxial reduction plate
OF: Occipitocervical fixation
BI: Basilar invagination
IAAD: Irreducible atlantoaxial dislocation
ADI: Atlantodental interval
CCA: Clivus canal angle
CL: Distance between the top of the odontoid process and the Chamberlain line
CMA: Cervicomedullary angle
JOA: Japanese Orthopaedic Association
CT: Computed tomography
MRI: Magnetic resonance imaging
NS: Not significant
